# Transforming echocardiography with artificial intelligence

**DOI:** 10.1007/s12471-025-01987-8

**Published:** 2025-09-05

**Authors:** Robert M. A. van der Boon, Jacco C. Karper

**Affiliations:** 1 Department of Cardiology, Cardiovascular Institute, Erasmus Medical Center, Rotterdam, The Netherlands; 2 Department of Cardiology, Wilhelmina Ziekenhuis, Assen, The Netherlands

**Keywords:** Artificial intelligence, Echocardiography, Cardiovascular disease

## Transforming cardiology with AI

Artificial intelligence is increasingly integrated into cardiovascular medicine, with new technologies entering clinical practice. This section provides a brief evaluation of recently available AI-driven products, reflecting the authors’ personal perspectives on their utility and limitations. The views expressed do not constitute an endorsement by the NVVC.

## Introduction

The demand for transthoracic echocardiography (TTE) continues to rise across all levels of care, driven by population ageing, broader indications, and the growing reliance on imaging in cardiovascular medicine. Simultaneously, studies have become more complex, requiring integration of advanced techniques such as strain and three-dimensional imaging, and the synthesis of multiple parameters for nuanced diagnoses, including diastolic dysfunction, complex valve disease and cardiac amyloidosis. These developments, combined with a shrinking workforce of trained sonographers, threaten the long-term accessibility of echocardiographic services.

In response, Erasmus Medical Center Rotterdam and Wilhelmina Ziekenhuis Assen have implemented Us2.AI, a CE-marked artificial intelligence (AI) based platform that automatically analyses echocardiographic images and generates structured, guideline-compliant reports. Here, we describe our experience in both clinical settings and reflect on its potential to improve the efficiency, scalability, and consistency of echocardiographic services.

## Technical features and capabilities

**Fig. 1:**
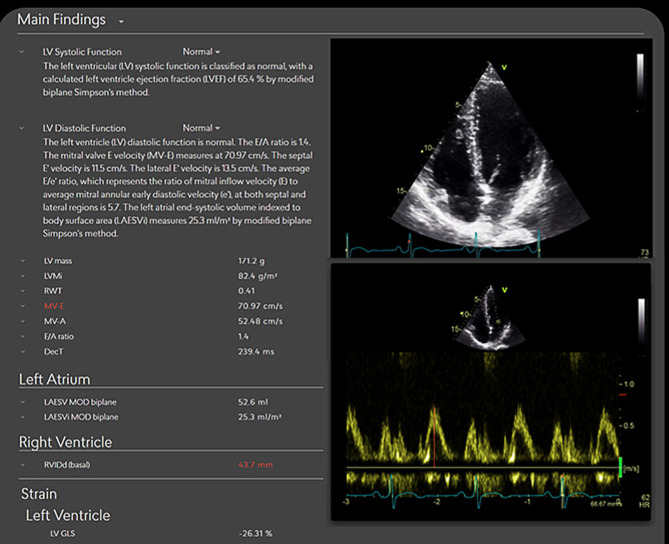
*AI-generated echocardiography report using Us2.AI.* Example of an automated report displaying measurements for systolic and diastolic function, left atrial volume, right ventricular size, and strain, with editable contours and Doppler tracings. All outputs are presented in a guideline-aligned format, allowing clinician review and adjustment

Us2.AI is a CE-marked, vendor-neutral software platform for automated analysis of TTE’s (Fig. 1). It integrates with standard ultrasound systems or with picture archiving and communication systems (PACS) and can be deployed either on-premises or in the cloud, depending on institutional requirements for data governance and infrastructure. The system performs automated view classification, image quality assessment, segmentation, and quantification of 68 guideline-recommended parameters, including left and right ventricular volumes and function, atrial size, Doppler indices (e.g., E/e’), and global longitudinal strain [1–3]. A full analysis and structured draft report are typically generated within two minutes after DICOM upload, without any manual input. Measurements are already generated during the echo examination, giving the sonographers live feedback on the generated analyses. All outputs, including numeric values, contours, and textual interpretations, are presented in an editable, ASE/EACVI-aligned format. Users can review, adjust, or reject results, with all changes automatically propagated throughout the report. This ensures full clinical control, traceability, and transparency in every step of the workflow. Integration with hospital systems is supported via HL7 or PDF, among other options. An automated audit trail is incorporated, allowing careful post-analysis follow-up for any necessary future evaluations of system performance.

In a multicenter validation study of 602 echocardiographic exams, the algorithm achieved an individual equivalence coefficient (IEC) < 0 for all parameters, with 95% confidence bounds < 0.25, indicating that AI-human variability was lower than expert inter-reader variability [1]. ICCs ranged from 0.74 for left ventricular ejection fraction to 0.97 for interventricular septum thickness. These findings support its reproducibility and readiness for clinical use.

## Potential clinical applications

The primary application of Us2.AI in both academic and general hospital settings is the automation of routine echocardiographic studies. These include indications such as hypertension, atrial fibrillation, heart failure, and follow-up of valvular or oncologic patients. In a prospective trial, AI reduced analysis time by over 70% (159 ± 66 vs. 325 ± 94 s, p < 0.01), while improving consistency in serial follow-up [4].

At Erasmus MC, although a large proportion of referrals are complex, the overall demand for echocardiography is rising, particularly in structured follow-up programmes for cardio-oncology, valve disease, and genetic screening. Here, Us2.AI is used to manage high-volume pathways, ensuring reproducibility while freeing expert staff to focus on more nuanced studies. In Wilhelmina Ziekenhuis Assen, with a greater proportion of standard indications, the system supports a broader share of the daily workflow. By fully automating acquisition-to-reporting, it mitigates workforce pressure and sustains throughput without compromising quality. In addition, AI-guided echocardiography may support earlier recognition of less common conditions in these settings, such as cardiac amyloidosis.

## Clinical considerations

Us2.AI can be installed on-premises or via a secure cloud infrastructure, depending on local policies. For both use cases, an on-premises solution was selected. Key requirements include DICOM routing, server capacity, and a secure browser-based access. Deployment at both hospitals was vendor-supported, with local and remote configuration and integration into the local infrastructures.

Integration into existing workflows is essential. Review and editing of AI-generated contours within familiar interfaces have been crucial to acceptance. This “human-in-the-loop” model preserves accountability while enabling efficiency. Training of sonographers, residents, and reporting cardiologists is critical, not only for the appropriate use and oversight of the system but also to prepare future echocardiography technicians to work confidently with AI-assisted analysis and to interpret algorithmic output critically. Post-deployment, regular quality monitoring is recommended to detect drift and build trust. Despite strong technical validation, further studies are needed to evaluate the impact on clinical outcomes, diagnostic accuracy, and, importantly, the adoption by caregivers and performance across various populations.

## Future perspectives

AI platforms such as Us2.AI may transform echocardiography across the diagnostic continuum. While automated reporting relieves a major bottleneck, it is only one part of a broader workflow. Future strategies must include upstream clinical decision support to guide appropriate use, demand-driven echo protocols and downstream innovations such as robotic acquisition, handheld imaging, and AI-supported triage in community settings.

If implemented thoughtfully, these technologies could shift echocardiography from a resource-intensive test to a scalable and standardized point-of-care diagnostic tool, reproducible and accessible far beyond the echo lab. Realizing this shift will require not only technical advancement but also clinician trust, clear governance, and ongoing research.
